# Visual Field Loss in Patients With Diabetes in the Absence of Clinically-Detectable Vascular Retinopathy in a Nationally Representative Survey

**DOI:** 10.1167/iovs.19-28063

**Published:** 2019-11

**Authors:** Yicheng K. Bao, Yan Yan, Mae Gordon, Janet B. McGill, Michael Kass, Rithwick Rajagopal

**Affiliations:** 1 Department of Medicine, University of Missouri–Kansas City School of Medicine, Kansas City, Missouri, United States; 2 Division of Public Health Sciences, Department of Surgery, Washington University School of Medicine, St. Louis, Missouri, United States; 3 Department of Ophthalmology and Visual Sciences, Washington University School of Medicine, St. Louis, Missouri, United States; 4 Division of Metabolism, Endocrinology and Lipid Research, Department of Medicine, Washington University School of Medicine, Missouri, United States

**Keywords:** diabetic retinopathy, frequency doubling technology, visual field, NHANES

## Abstract

**Purpose:**

Neuroretinopathy is increasingly being recognized as an independent cause of vision loss in diabetes. Visual field loss, as detected by frequency doubling technology (FDT)-based visual perimetry, is a sign of neuroretinopathy and occurs in early stages of diabetic retinopathy (DR). Here, we hypothesized that FDT visual field testing could identify patients with diabetic neuroretinopathy in the absence of clinically detectable microvascular DR.

**Methods:**

All National Health and Nutrition Examination Survey (NHANES) 2005–2008 participants receiving fundus photography and visual field screening by FDT were included in this study. Participants with self-reported glaucoma, use of glaucoma medications, or determination of glaucoma based on disk features were excluded. Visual fields were screened using FDT protocol in which participants underwent a 19-subfield suprathreshold test.

**Results:**

Patients with diabetes but no DR were more likely to have ≥1 subfield defects at 5%, 2%, and 1% probability levels than patients without diabetes (41.3% vs. 28.6%; 27.4% vs. 17.5%; 15.9% vs. 9.4%; all *P* < 0.0008). Multivariable regression showed that each additional glycated hemoglobin % (HbA1c) was associated with 19% greater odds of having ≥1 visual subfield defects in those with diabetes without DR (odds ratio: 1.19, 95% confidence interval: 1.07–1.33; *P* = 0.0020).

**Conclusions:**

Patients with diabetes have visual field defects in the absence of clinically detectable DR, suggesting neuroretinopathy precedes classical microvascular disease. These defects become more frequent with the onset of visible retinopathy and worsen as the retinopathy becomes more severe. Longitudinal studies are required to understand the pathogenesis of diabetic neuroretinopathy in relation to classic DR.

Diabetic retinopathy (DR), a common microvascular complication of diabetes, is directly related to plasma glucose levels. In fact, current diagnostic criteria for diabetes established by the American Diabetes Association (glycated hemoglobin [HbA1c] ≥ 6.5% or fasting plasma glucose ≥ 126 mg/dL) are based on plasma glucose levels associated with the incidence of moderate DR in large, population-based studies.^1^ Characteristic microvascular lesions and capillary leakage are the hallmarks of DR. Contemporary treatments of this disease, including laser photocoagulation, corticosteroids, and even antagonists of the vascular endothelial growth factor (VEGF) pathway, target late stages of the disease and may be ineffective in up to one third of patients. In addition, chronic anti-VEGF therapy is associated with deleterious effects in clinical settings and in rodent models.^2^ Therapies that target the disease process at earlier stages in the diabetic eye may improve public health.

In preclinical studies and small clinical analyses, functional visual deficits in diabetes are observed prior to vascular retinopathy.^3^–^6^ These deficits are collectively termed diabetic neuroretinopathy, and their relationship to classical DR is incompletely understood. Thinning of the inner retina during diabetes can be detected by optical coherence tomography, but changes are often subtle and progressive much like loss of the optic nerve head rim due to glaucoma. But despite being challenging to detect, identification of diabetic neuroretinopathy could present an opportunity for prevention of the more vision-threatening vascular lesions in the diabetic retina. Visual field testing using frequency doubling technology (FDT) was introduced as a rapid screening tool to identify retinal ganglion cell loss (specifically of the magnocellular variety) in early glaucoma,^7^ but it has also shown some utility in detecting damage in early DR.^3^,^8^ Here, we hypothesized that diabetes itself (without DR) causes inner retinal visual defects, and that FDT visual field testing reveals such defects. We report findings from a secondary analysis of a large cohort representative of the US noninstitutionalized civilian population showing that patients with diabetes, but no clinically detectable DR, have significant FDT-detected field loss and that these patterns of field loss worsen during the course of classical DR.

## Methods

### Study Population

**Figure 1:**
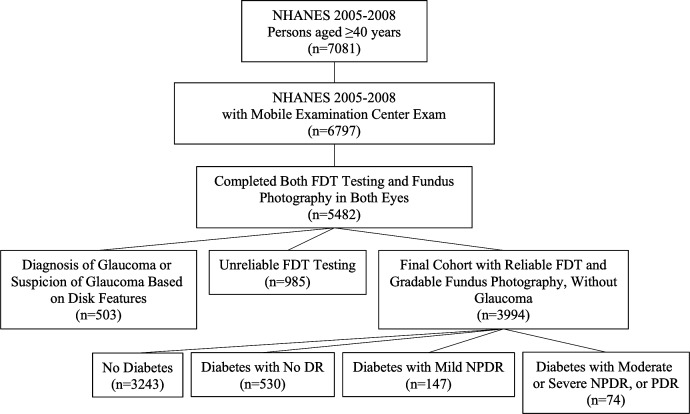
Cohort selection flowchart for NHANES 2005–2008 Participants Aged 40 Years and Older With Fundus Photography and Frequency Doubling Technology Visual Field Testing.

The study population was gathered from the National Health and Nutrition Examination Survey (NHANES) 2005–2008, which used a stratified, multistage probability design to allow for representation of the US noninstitutionalized civilian population.^9^ This work was conducted in compliance with the Declaration of Helsinki. Cohort selection for this study is depicted in Figure 1. All participants receiving both fundus photography (with grading) and FDT visual field testing were eligible for inclusion in this study. Participants with a self-report of glaucoma diagnosis or determination of “possible,” “probable,” or “definite glaucoma” by three glaucoma specialists at Johns Hopkins University based on disk features on fundus photography (such as vertical cup-to-disk ratio ≥ 0.6, disk hemorrhage, excavation, notch, and tilt), were excluded.

Retinopathy scores were extracted from grading of 2-field nonmydriatic photography of each eye using modified Early Treatment of Diabetic Retinopathy Study (ETDRS) criteria, as described previously.^10^,^11^ Diabetes mellitus was defined as present if any of the following criteria were met: (1) self-report of diabetes in the NHANES questionnaire; (2) HbA1c ≥ 6.5%; or (3) use of oral or injectable medicines for hyperglycemia. Protocols for NHANES visual acuity testing have been described.^12^ For this analysis, best-corrected Snellen-equivalent vision was defined as the better of either (1) presenting visual acuity with current lens prescription or (2) visual acuity after NHANES-conducted objective refraction.

Visual fields were evaluated in a subset of NHANES participants aged 40 years and older using FDT perimetry screening procedures.^13^,^14^ Participants underwent a 19-subfield suprathreshold screening test in two replicates (conducted on the same day) using the N-30-5 test on the Humphrey Matrix FDT (Carl Zeiss Meditec, Dublin, CA, USA). Stimuli were presented at the age-adjusted 5% probability level. If this was missed, a target was presented at the 2% probability level, and if missed, it was presented at the 1% probability level. A test was considered unreliable if the false positive rate was greater than 33%, or if there were greater than 33% fixation losses by physiologic blind spot testing, or if the technician noted any fixation error.^12^ NHANES used a stringent 2-2-1 algorithm to define visual field loss by FDT testing.^15^ Adopting these standards, patients in this study were considered to have visual field loss if they had a defect in at least two subfields on both the first test and the second test, and if at least one of those subfields were defective in both tests. In addition to 2-2-1 algorithm-based visual field loss, the mean number of defective visual fields for each eye per patient was calculated for each of the three threshold levels (5% or lesser, 2% or lesser, and 1%) using data from both replicates; the mean number of subfield defects between both trials were calculated for each eye and summed to calculate the total number of defects across both eyes.

### Statistical Analysis

Participants were placed in four groups based on diabetes status and retinopathy severity: (1) no diabetes, (2) diabetes with no DR, (3) diabetes with mild nonproliferative DR (NPDR), and (4) diabetes with moderate or severe NPDR, or proliferative DR (PDR). Baseline characteristics between these groups were compared using ANOVA for continuous variables and Rao-Scott χ^2^ test for categorical variables. The number of participants in each group with 2-2-1 algorithm defined visual field loss or ≥1, 5, 10, or 15 subfield defects was compared with Rao-Scott χ^2^ test. Mean number of subfield defects was compared across all four groups with ANOVA while the mean number of subfield defects between the diabetes and diabetes with no retinopathy groups was compared with Hochberg's GT2 post hoc test. Agreement between number of subfield defects between eyes was compared using Cohen's κ. Multivariable logistic regression was used to evaluate the association between covariates and the presence of visual field defects in participants without retinopathy. Lipids were not included in the multivariable model, because those values were only drawn in the fasting group, resulting in missing data for half of the cohort. Sampling weights provided by the National Center for Health Statistics, Centers for Disease Control and Prevention were used. A 2-tailed *P*-value of <0.05 was considered significant. All data were analyzed using SAS 9.4 (2012, SAS Institute Inc., Cary, NC, USA).

## Results

During 2005–2008, NHANES collected information on 20,497 patients and captured fundus photos and FDT fields on 5651 of them. Among these, 5482 patients had gradable photos in both eyes and were eligible for this study. After excluding 503 patients for suspicion of glaucoma (or definite diagnosis of glaucoma) and 985 patients for unreliable FDT testing, 3994 participants and 7988 eyes were included in the final analysis. Adjusting for survey weighting design, this sample represents 85,656,498 people in the United States and were 47.1% male, 78.4% Caucasian, 8.7% African American, 5.4% Mexican American, 7.6% other ethnicities, with a mean age of 55.7 years (SEM: 0.41). Participants with diabetes (with and without retinopathy) were older, more likely not to be Caucasian, had higher HbA1c, greater levels of dyslipidemia, and elevated C-reactive peptide (CRP) compared with patients without diabetes. Notably, there were no differences in the mean arterial blood pressure between groups (Table 1).

**Table 1:** Characteristics of the NHANES 2005–2008 Participants Aged 40 Years and Older With Fundus Photography and FDT Visual Field Testing, by Diabetes Status and Retinopathy Severity

	**DM−**	**DM+ DR−**	**DM+ Mild NPDR**	**DM+ Moderate, Severe NPDR, PDR**	P*
*n*	3243	530	147	74	
Mean age, y (95% CI)	54.8 (54.1–55.6)	59.7 (58.3–61.1)	61.4 (59.7–63.1)	58.7 (55.1–62.2)	<0.0001
Sex (%)†					0.1301
Male	1606 (46.8)	247 (46.2)	87 (56.4)	41 (54.8)	
Female	1637 (53.2)	283 (53.8)	60 (43.6)	33 (45.2)	
Ethnicity (%)†					<0.0001
Caucasian	1914 (80.6)	224 (66.7)	53 (59.3)	16 (47.9)	
African American	535 (7.3)	143 (15.5)	49 (22.2)	33 (32.0)	
Mexican American	481 (4.9)	103 (7.7)	31 (8.8)	20 (14.5)	
Other	313 (7.2)	60 (10.1)	14 (9.7)	5 (5.6)	
HbA1c, % (95% CI)	5.41 (5.39–5.43)	6.98 (6.79–7.17)	7.81 (7.43–8.20)	8.35 (7.55–9.15)	<0.0001
MABP, mm Hg (95% CI)	89.7 (89.2–90.2)	90.5 (88.6–92.4)	88.4 (85.9–90.9)	89.8 (85.9–93.7)	0.5119
CRP, mg/dL (95% CI)	0.42 (0.37–0.46)	0.69 (0.54–0.83)	0.51 (0.39–0.62)	0.67 (0.51–0.84)	0.0089
Triglycerides, mg/dL (95% CI)	141.2 (135.4–147.0)	191.6 (150.2–233.0)	180.0 (134.3–225.6)	146.3 (97.4–195.2)	<0.0001
LDL, mg/dL (95% CI)	122.0 (119.7–124.3)	104.3 (98.5–110.2)	101.1 (86.9–115.3)	111.9 (88.2–135.7)	<0.0001
HDL, mg/dL (95% CI)	54.9 (54.2–55.7)	47.2 (45.6–48.8)	48.8 (46.7–50.8)	48.0 (43.2–52.9)	<0.0001
Cholesterol, mg/dL (95% CI)	206.9 (205.2–208.6)	192.5 (187.3–197.7)	185.5 (172.3–198.7)	190.4 (166.9–213.8)	<0.0001

DM, diabetes mellitus; DR, diabetic retinopathy; NPDR, nonproliferative diabetic retinopathy; PDR, proliferative diabetic retinopathy; MABP, mean arterial blood pressure; CRP, C-reactive peptide; LDL, low-density lipoprotein; HDL, high-density lipoprotein.

* *P* values were calculated using ANOVA for continuous variables and the Rao-Scott χ^2^ test for categorical variables.

† Percentages are survey design adjusted.

Participants with diabetes and no DR were more likely than those without diabetes to have 2-2-1 algorithm defined visual field loss (Table 2; 8.2% vs. 4.4%; *P* < 0.0001). Participants with diabetes and moderate or severe NPDR or PDR were more likely than all groups to have 2-2-1 algorithm defined visual field loss (24.0% vs. 9.3% vs. 8.2% vs. 4.4%; *P* < 0.0001; Table 2). Participants with diabetes but no clinically detectable retinopathy were more likely to have a total of ≥1 subfield defects at the <5%, 2%, and 1% probability levels than patients without diabetes (all *P* < 0.05; Table 2). In sensitivity analyses, participants with diabetes and no retinopathy were more likely to have ≥10 subfield defects at all threshold levels compared with patients without diabetes (all *P* < 0.05; Table 2) and were also more likely to have ≥15 defects at the 2% and 1% test thresholds, which were the most stringent (all *P* < 0.05; Table 2). Participants with diabetes and no retinopathy had no significant differences in the number of subfield defects compared with participants with mild NPDR. However, participants with diabetes and moderate or severe NPDR, or PDR had more subfield defects than all other groups (Table 2; *P* < 0.0001). Patients with no diabetes had a mean of 1.9 (95% confidence interval [CI]: 1.7–2.2) subfield defects at the 5% probability level, whereas those with diabetes and no DR had a mean of 3.1 (95% CI: 2.3–3.9) subfield defects (*P* = 0.0019), whereas those with diabetes and mild NPDR had 3.5 (95% CI: 1.7–5.3) subfield defects, and those with diabetes, moderate or severe NPDR, or PDR had a mean of 7.0 (95% CI: 4.8–9.2) subfield defects at the <5% probability level (*P* < 0.0001). There was no difference between the mean number of subfield defects at the 2% and 1% probability levels comparing patients without diabetes with those with diabetes but no retinopathy (*P* = 0.1678, *P* = 0.3585; Table 2). Participants showed substantial agreement between eyes demonstrating ≥1 defects at the 5% threshold level (κ = 0.6030). Seven hundred thirty-six (18.4%) participants had defects in one eye only at the 5% threshold level across both FDT test replicates. Participants showed substantial agreement between two tests demonstrating ≥1 defects at the 5% threshold level in both the right eye (κ = 0.6504) and left eye (κ = 0.7046).

**Table 2:** Number of Participants With 2-2-1 Algorithm Defined Visual Field Loss, or ≥1, 5, 10, or 15 Visual Subfield Defects at Each Threshold Level by Diabetes Status and DR Severity, NHANES 2005–2008 Participants Aged 40 Years and Older With Fundus Photography and FDT Visual Field Testing

	**DM−**	**DM+ DR−**	P*	**DM+ Mild NPDR**	**DM+ Moderate, Severe NPDR, PDR**	***P*****†**
Number of participants with any field loss as determined by 2-2-1 algorithm	208 (4.4)	49 (8.2)	<0.0001	19 (9.3)	19 (24.0)	<0.0001
<5% threshold						
Number of field defects, *n* (%)						
≥1	1039 (28.6)	214 (41.3)	<0.0001	66 (40.4)	46 (64.7)	<0.0001
≥5	488 (11.7)	113 (19.7)	0.0003	40 (23.8)	31 (36.1)	<0.0001
≥10	276 (6.9)	66 (10.9)	0.0093	23 (11.4)	23 (22.7)	<0.0001
≥15	184 (4.4)	34 (5.5)	0.3961	14 (7.2)	17 (16.4)	0.0034
Mean field defects (95% CI)	1.9 (1.7–2.2)	3.1 (2.3–3.9)	0.0019	3.6 (1.7–5.4)	6.8 (4.7–9.0)	<0.0001
<2% threshold						
Number of field defects, *n* (%)						
≥1	664 (17.5)	151 (27.4)	0.0001	48 (26.6)	38 (47.8)	<0.0001
≥5	260 (5.6)	59 (9.7)	0.0005	23 (11.9)	22 (22.2)	<0.0001
≥10	141 (3.1)	32 (5.3)	0.0376	12 (6.6)	17 (16.5)	<0.0001
≥15	81 (1.7)	19 (3.6)	0.0281	8 (3.8)	12 (10.4)	<0.0001
Mean field defects (95% CI)	1.0 (0.8–1.1)	1.7 (1.1–2.4)	0.1678	1.8 (0.7–2.9)	4.6 (2.8–6.3)	0.0012
<1% threshold						
Number of field defects, *n* (%)						
≥1	384 (9.4)	93 (15.9)	0.0008	34 (19.1)	26 (31.8)	<0.0001
≥5	134 (2.9)	25 (4.3)	0.1593	8 (3.8)	15 (12.8)	0.0010
≥10	62 (1.2)	15 (2.9)	0.0229	7 (3.6)	10 (9.2)	<0.0001
≥15	37 (0.7)	11 (2.3)	0.0059	4 (1.2)	8 (6.6)	<0.0001
Mean field defects (95% CI)	0.5 (0.4–0.5)	0.9 (0.4–1.4)	0.3585	0.8 (0.2–1.4)	2.5 (1.4–3.6)	0.0038
Best visual acuity			0.1260			0.0656
None (20/20–20/40)	3126 (99.5)	505 (98.8)		138 (98.9)	63 (88.7)	
Moderate (20/50–20/80)	29 (0.4)	6 (1.1)		4 (1.1)	5 (11.3)	
Severe (≥20/200)	3 (0.1)	1 (0.1)		0 (0)	0 (0)	

All percentages are survey design adjusted. DM, diabetes mellitus; DR, diabetic retinopathy; NPDR, nonproliferative diabetic retinopathy; PDR, proliferative diabetic retinopathy.

* *P* values comparing DM− and DM+ DR− groups reported from Rao-Scott χ^2^ test for categorical variables and Hochberg's GT2 post hoc test for continuous variables.

† *P* values comparing all four groups calculated reported from Rao-Scott χ^2^ test for categorical variables and ANOVA for continuous variables.

In participants without DR, a multivariable logistic regression model adjusting for age, sex, ethnicity, and CRP showed that each additional HbA1c percentage point was associated with a 19% increase in odds of having ≥1 visual subfield defect at the 5% level (odds ratio [OR]: 1.19, 95% CI: 1.07–1.33; *P* = 0.0020; Table 3). African American ethnicity (OR: 2.23, 95% CI: 1.75–2.85; *P* < 0.0001) and Mexican American ethnicity (OR: 1.80, 95% CI: 1.26–2.59; *P* = 0.0023) and older age were significantly associated with odds of ≥1 visual subfield defect.

**Table 3:** Multivariable Logistic Regression Model For Odds of ≥1 Visual Subfield Defects at 5% Level in Patients Without DR, NHANES 2005–2008 Participants Aged 40 Years and Older With Fundus Photography and FDT Visual Field Testing

	**Adjusted OR (95% CI)**	***P***
HbA1c	1.19 (1.07–1.33)	0.0020
CRP	0.97 (0.84–1.12)	0.6725
Sex		0.0538
Male	1.23 (0.99–1.53)	
Female	Reference	
Age, y		
40–49	Reference	
50–59	1.25 (0.93–1.68)	0.1388
60–69	1.70 (1.25–2.30)	0.0013
≥70	3.21 (2.47–4.18)	<.0001
Ethnicity		
Caucasian	Reference	
African American	2.23 (1.75–2.85)	<0.0001
Mexican American	1.80 (1.26–2.59)	0.0023
Other	1.53 (1.04–2.26)	0.0313

Other metabolic parameters related to type 2 diabetes were investigated for their correlation with visual field defects. In multivariable logistic regression models controlling for age, sex, ethnicity, CRP, and HbA1c, triglycerides (mg/dL) (OR: 1.00, 95% CI: 0.99–1.00; *P* = 0.2694), low density lipoprotein (mg/dL) (OR: 1.00, 95% CI: 0.99–1.01; *P* = 0.8624), total cholesterol (mg/dL) (OR: 1.00, 95% CI: 0.99–1.00; *P* = 0.4212), and homeostatic model for insulin resistance (OR: 0.98, 95% CI: 0.94–1.03; *P* = 0.4364) were not found to be significant predictors of having ≥1 visual subfield defect at the 5% level. Duration of diabetes was also found to not be a significant predictor (per year OR: 1.00, 95% 0.99–1.00; *P* = 0.5687).

**Figure 2:**
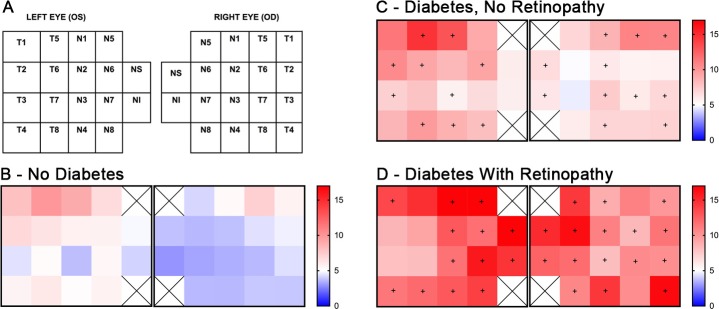
Patterns of visual field loss in diabetes and DR. Frequency-doubling technology-based visual field screening protocol used in NHANES consisted of 19 subfields as depicted (A). Percentage of participants with field loss in the indicated subfields among the group without diabetes (B), the group with diabetes but without visible retinopathy (C), and the group with visible DR (D). + denotes Rao-Scott χ^2^ test P < 0.05 between cohorts B versus C and cohorts B versus D.

Between groups, a comparison of field loss across subfields of the FDT perimeter (Fig. 2A) showed differences in both the frequency of loss within various subfields and in the overall patterns of field loss. Individuals with DR had the highest percentage of participants with a field defects in several of the 19 subfields tested, compared with patients with no retinopathy (Figs. 2B, 2D). Notably, these defects tended to occur symmetrically and preferentially in the nasal visual fields (Fig. 2D). Participants with diabetes and no microvascular lesions had higher frequencies of field loss when compared with those without diabetes, and the defects in this group showed a diffuse pattern (Fig. 2C).

## Discussion

This post hoc analysis of a large-scale, national, population-based survey demonstrates that compared with healthy controls, FDT visual field defects occur more frequently in participants with diabetes in the absence of outright retinopathy, suggesting an inner retinal sensory neuropathy associated with diabetes itself. These defects become more frequent with the onset of visible retinopathy and worsen as the retinopathy becomes more severe. Furthermore, visual field defects associated with DR tend to occur in the nasal subfields.

One interpretation of these data is that diabetes induces a sensory neuropathy of the retina, similar to peripheral neuropathy in diabetes, preceding the onset of visible retinal microvascular disease. This notion is supported by other studies using structural and functional analyses of patients with diabetes but no retinopathy. It is possible we detected early glaucomatous loss in patients with diabetes, but two features of our study argue against this conclusion. First, patients with established glaucoma, use of glaucoma medications, or funduscopic evidence of glaucoma or suspicion of glaucoma, based on the standards set by NHANES, were excluded from the study. Second, the visual defects in patients with diabetes are related to the severity of DR—a feature not consistent with glaucomatous field loss.

Neural deficits occur in patients with diabetes and no clinically significant DR. Jackson et al.^3^ describe a 2.9-dB loss in central sensitivity in a group of 23 patients with diabetes (48% type 1 diabetes) without NPDR compared with a control group without diabetes using FDT (*P* = 0.0002). Parravano et al.^4^ found that patients with type 1 diabetes and no retinopathy had more Humphrey Matrix perimetry defects when compared with controls. These previous clinical studies using perimetry testing in diabetes included high proportions of patients with type 1 disease. In contrast, 95% of the population included in this study is estimated to have type 2 diabetes, reflective of the surveyed population.^16^ Therefore, our findings are novel in describing FDT visual field loss in a predominantly type 2 diabetes population without retinopathy.

Mechanisms underlying vision loss in patients with diabetes without DR are poorly understood. Sohn et al.^6^ reported thinning of the nerve fiber layer, ganglion cell layer, and inner plexiform layer as measured by optical coherence tomography prior to the onset of microvascular disease in their longitudinal study of 45 patients with diabetes. Diabetes is also associated with histologic evidence of retinal ganglion cell apoptosis.^17^,^18^ In experimental models of diabetes, the retina shows reduced responsivity to insulin,^19^ and therefore diabetes-associated retinal thinning and neural loss could be explained by loss of an important source of neurotrophic support.

A caveat to this study is that the cross-sectional nature of NHANES survey data limits attempts to draw conclusions on causal pathophysiology. Although this study shows functional visual deficits associated with diabetes in the absence of overt retinopathy, subclinical microvascular lesions—undetectable by current imaging—may be responsible. This study cannot definitively conclude whether diabetic neuroretinopathy or DR occur in parallel or in sequence. However, strengths of the present analysis include a large sample size, nationally representative sample, independent grading of fundus photography, and high reliability of visual field data acquired.

In summary, these data demonstrate significant inner neuroretinopathy in diabetes occurring in the absence of typical microvascular lesions. Longitudinal studies are required to understand the pathogenesis of diabetic neuroretinopathy and classic DR.
